# Towards Industrially Important Applications of Enhanced Organic Reactions by Microfluidic Systems

**DOI:** 10.3390/molecules29020398

**Published:** 2024-01-13

**Authors:** Ayesha Zafar, China Takeda, Asif Manzoor, Daiki Tanaka, Masashi Kobayashi, Yoshitora Wadayama, Daisuke Nakane, Adnan Majeed, Muhammad Adnan Iqbal, Takashiro Akitsu

**Affiliations:** 1 Department of Chemistry, Faculty of Science, University of Agriculture, Faisalabad 38040, Pakistan; 2 Department of Chemistry, Faculty of Science, Tokyo University of Science, Tokyo 162-8601, Japan; 3 Research Organization for Nano & Life Innovation, Waseda University, Tokyo 169-8050, Japan

**Keywords:** microfluidic system, droplet synthesis, microfluidic organic synthesis, organic compounds

## Abstract

This review presents a comprehensive evaluation for the manufacture of organic molecules via efficient microfluidic synthesis. Microfluidic systems provide considerably higher control over the growth, nucleation, and reaction conditions compared with traditional large-scale synthetic methods. Microfluidic synthesis has become a crucial technique for the quick, affordable, and efficient manufacture of organic and organometallic compounds with complicated characteristics and functions. Therefore, a unique, straightforward flow synthetic methodology can be developed to conduct organic syntheses and improve their efficiency.

## 1. Introduction

Many researchers have concentrated on manufacturing metal complexes, including proteins, that may be useful for the development of new drugs or fuel cells [1].

With the proliferation of microelectromechanical systems (MEMS) technology, the syntheses of microfluidic devices or microdroplets have become increasingly important as tools for chemical analysis and synthesis [2,3]. Microfluidic devices (microreactors, microchemical chips, etc.) that integrate the functions of chemical synthesis, analysis equipment, and chemical plants in a compact form are being investigated [4,5,6,7]. By applying semiconductor integrated circuit microfabrication technology using MEMS, chemical and biochemical analysis tools have become more compact with high performance. Moreover, micro/nanofluidic devices of approximately 10^−9^ to 10^−12^ L for chemical synthesis and cell function analysis have been fabricated [8,9]. The reagent reacts by pouring the solution into a groove cut on a small plate, and the flow path is designed to allow the reaction reagents, products, and waste liquids to meet and separate [10]. Here, MEMS refers to ultra small devices with a total length of less than less than a millimeter, in which mechanical parts, sensors, electronic circuits, etc., are integrated into silicon substrates, glass substrates, organic materials, etc. [11].

Zare et al. generated microdroplets in a spray-like manner to elucidate the reaction kinetics of organic synthesis at the microscale level. For example, the reaction rate for the Pomerantz–Fritsch synthesis is 100,000 times faster than the beaker scale [12]. Microfluidic devices are also capable of extraction operations. For example, Craig et al. reported the application of organic solvent extraction for Cu^2+^ ions from aqueous solutions [13]. Tuck et al. explained the improvement in microscale chemical reaction rates regarding Gibbs energy. Using the oligomer polymerization of amino acids, they reported that the Gibbs energy, which is positive at the beaker scale, becomes negative at the microscale, promoting spontaneous reactions [14].

The advantages of chemical synthesis using microfluidics are as follows: (1) Owing to their small size, only small quantities of materials that are difficult to obtain or expensive can be processed. Smaller sample volumes are advantageous, particularly for analytical purposes. (2) The reaction is either completed immediately after the rapidly flowing solutions meet or is limited to a short time when the solutions are in contact with each other. This is because the distance over which the substances can spread is limited to an extremely short range. (3) High functionality may require less effort (isolation, purification, and sequential reactions) than synthesis in a beaker. By devising the groove design of the microfluidic device, it is possible to separate product AB from the unreacted products, react product AB with reactant C, or separate the target ABC from unreacted AB or unreacted products. Therefore, refinement from C is not required [15,16]. (4) Space savings are evident when compared to the installation space of reaction vessels, draft columns, etc., in a typical chemical laboratory. (5) The lower cost is related to (1), but because the amount of reagents required (often with higher yields than conventional methods), solvents, and thermal energy (heating time) are reduced, less liquid waste is generated. The amount of spent reagent is reduced (disposal costs), which is both economically and environmentally beneficial [17]. Kim et al. devised a microfluidic approach to chemo selectively functionalize the ortho position of iodophenyl carbamates via an extremely rapid anionic Fries rearrangement [18].

A research team prepared and developed single crystals of Schiff base complexes containing rare elements in organic solvents. There is always a risk of volatile solvent leakage because crystals are formed when the organic solvent in the solution volatilizes. Moreover, environmental measurements in laboratories may reveal health and fire risks. Fumes released by volatilizing organic solvents in laboratories are dangerous to human health. In this case, expertise or knowledge regarding the interfacial organic solvents utilized for crystallization (two layers in a test tube) and preparation (mutual solute and solvent dissolution via vapor diffusion) may be crucial for the experimental setup of the microfluidic preparation apparatus. In light of this correlation, this paper presents chemistry using interfacial organic solvents obtained from existing experiments. In addition, artificial metal proteins containing these metal complexes have recently attracted attention, and the handling of fragile biomolecules and materials using microfluidic devices is becoming active, overcoming the shortcomings of multi-step and long-term synthesis [10].

As a general introduction, we present some of the advantages of microfluidic synthesis along with research examples. First, compared with previous methods, microfluidic devices offer various advantages, including yield and separation.

The synthesis of various organic ligands, metal complexes, and protein complexes via high-throughput synthesis using a microfluidic device has been explained. Multi-step synthesis methods typically include the following steps.

Step 1: Synthesis of organic ligands from raw materials and reactants.

Step 2: Synthesis of the metal complex from organic ligands and metal sources.

Step 3: Addition of the protein to the complex and the protein containing the metal complex.

Y-shaped microfluidic devices have been designed to couple the product of a previous step to one of the reactants of the next step [10,15].

Concerning the reaction time, in a conventional reaction, the solution is stirred, but the laminar flows at the two inlets can still meet and react. Additionally, protrusions can be added to disrupt the flow and promote the mixing of laminar solutions. In addition to yield (2-fold) and purity (immediate and easy isolation), laminar flow has the remarkable advantages of low temperature and short reaction time. Metal complexes containing proteins are generally synthesized using traditional methods (i.e., in a beaker). However, existing methods have the potential to modify proteins, which are affected by organic solvents. These changes in proteins increase the difficulty of removing proteins carrying metal complexes from the reaction fluid [19].

Second, the microfluidic system is useful for the conversion of specific functional groups into hazardous products under harsh reaction conditions. Recently, microfluidic devices and chemistry-related reactions have been developed. Fast reaction rates and effective synthesis can be accomplished with these devices, while the period of diffusion of the chemical species is short in microscale channels [20]. Because of the laminar flow, many chemical species do not contribute to the reaction in devices with Y-shaped channel designs, despite their frequent use in easy manufacturing. This apparatus has been used for the bromination of organic substances. In a simple microchannel, in the absence of an iron catalyst, the monobromination of two or more bromo groups was achieved, but no addition was made to the organic compounds. Two simple solutions were considered, and initially, mixed particles of iron were utilized as reagents. Iron particles either blocked or flowed through the channel before the chemical reaction; however, dibromination was not observed. Alternatively, sputtering or vapor deposition can be used to cover the microchannel surface with an iron catalyst [21,22].

Conventional bromo group addition reactions are dangerous because of the toxicity and corrosiveness of bromine. In addition, the bromination reaction rate is usually low, and the reaction is considered complicated. In contrast, the specificity of the microscale reactor increased reaction speed and improved recovery rates of the target substances [23]. The highly toxic and corrosive bromo group addition reaction has been performed under highly efficient and nonhazardous conditions using a simple microfluidic method. The proposed silicon/glass structure prevents the leakage and circulation of internal and external gases, thereby eliminating the need for atmospheric control. Harmful bromine-containing gases are trapped in the isolated microchannels of the device [24,25].

Third, chemical reactions can be implemented by achieving specific physical conditions such as temperature (dissipation and activation), diffusion (mass transfer, solvent viscosity), and surface tension energy. Azo compounds are attractive materials for anticancer drugs and solar cells. However, existing synthesis methods are not efficient. For example, to eliminate heat produced during the reaction, the temperature is set at 0 °C [26]. Additionally, high concentrations of acidic and alkaline reagents cause product decomposition; therefore, the pH must be neutral. However, such complex chemical reactions are difficult to achieve using droplet or single-step synthetic reactions [27].

In addition, the existing method requires recrystallization because unreacted materials and byproducts are mixed. However, the microdroplet method ensures a high product purity, and additional recrystallization is not required in this method. This result consistently provides the high diffusion length of the chemical moieties contributed to the high-efficiency chemical reaction. These results showed that complex chemical synthesis was possible in microdroplets and that chemical reactions using microdroplets could spread chemical species quickly and efficiently [14].

In this way, microscopic water droplets were considered ‘little laboratories,’ and complex chemical azo compounds were synthesized for the first time. However, the synthesis of azo compounds requires precise pH control, and the reaction is exothermic. Synthesis using microdroplets has advantages over temperature control and does not require the pH reagent concentration, synthesis time, or recrystallization. The microdroplets quickly release heat without any heat-induced side reactions. Accurate pH control and high-purity synthesis are possible using the micro-liquid titration method [28,29]. Microfluidics is one of the most rapidly rising scientific topics. It has practical applications in all aspects of life, including the pharmaceutical business, diagnosis, chemical analysis, drug screening, medical care, environmental control systems, private testing, and food processing [30,31,32]. All of these advantages are not possible without the use of various pumping strategies for microfluidics. These shortcomings are compensated for by active pumping processes, which result in extremely efficient, robust, completely automated microfluidic flow regulation systems. These are outfitted with various pumps (electromagnetic, syringe, and magnetic) and valves to accurately control the fluid flow which make this microfluidic system costly. We anticipate that novel microfluidic systems will be designed to be as small as feasible, regardless of fluid flow control. The authors also believe that active flow control mechanisms will displace passive techniques and integrate all of the advantages of currently available methods, resulting in the development of revolutionary miniaturized high-precision all-in-one microfluidic systems with no external elements. We anticipate that current microelectronics research will greatly lower the size and power consumption of microfluidic devices. This fact will result in low-cost, portable, automated, and extremely precise systems that can perform a wide range of tests on a single chip [33,34]. Another reason behind limited use of microfluidic system at an industrial scale is that academic laboratories typically produce microfluidic devices utilizing materials and processes that require significant modification for industrial application, and they lack the time and resources to further develop these in-house. For the same reason, the industry may be hesitant to invest in early-stage innovations that have yet to be demonstrated to work outside of academic laboratories. To fulfill this above problem, recently, he Canadian government made a large investment in Precision Nanosystems, a local biotechnology company, for vaccine production using nanoparticle technology [35].

## 2. Industrial Applications and Microfluidic Synthesis

### 2.1. Pharmaceutical Applications

**Scheme 1:**
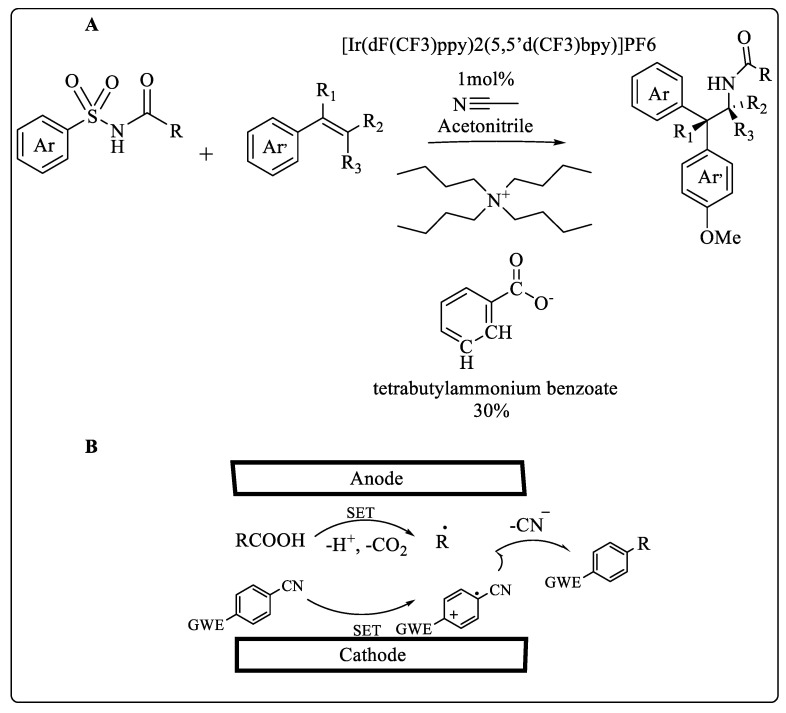
(**A**) Microfluidic droplet system enabled HTE reaction discovery (**B**) μRN-eChem-based decarboxylative arylation.

Photochemical reactions can be enhanced through the implementation of continuous flow technology. Scientists have developed a High Throughput Experimentation (HTE) droplet microfluidic platform that enables high-throughput drug discovery in flow to generate pharmaceutically relevant compound libraries. It is possible to handle several samples simultaneously over a long period by segmenting them with an immiscible phase [36,37]. Greater alkene compatibility and enhanced reactivity were observed with pharmacologically relevant sulfonyl acetamides containing heteroarenes, as shown in Scheme 1A. The in-droplet reaction discovery screen enabled researchers to expand upon their previously reported alkene amino arylation methodology to include substrates with increased structural complexity. It also allowed them to identify reactivity trends and [29] structure–activity relationships to inform ongoing mechanistic investigations [38]. The present study introduces a microfluidic redox-neutral electrochemistry platform (µRN-eChem), which is widely applicable to single-electron transfer chemistry (SET), such as radical–radical cross-coupling, Minisci reactions, and nickel-catalyzed C(sp^2^)-O cross-couplings (Scheme 1B). A microfluidic channel facilitates selective transformation by accelerating molecular diffusion across a cathode and anode simultaneously while outpacing the decomposition of intermediates. Due to the excellent conductivity of the microfluidic channel, no additional electrolyte was required. An electrosynthesis of a nematic liquid crystal compound was demonstrated using µRN-eChem in two steps [39].

An isoporous nanostructured membrane is incorporated into microfluidics-integrated microscale organic electrochemical transistors (OECTs). These label-free devices were used to cure Alzheimer’s disease (AD) by detecting Aβ protein aggregates in human serum with a performance exceeding those of several other systems [40,41]. To capture protein aggregates, Congo Red (CR) molecules were functionalized on the membrane. During the accumulation process, the membrane surface’s capacitance changed, modulating the gate voltage felt by the transistor channel. Microfluidic channels served as immunoreaction chambers that reduced analytical time compared to microwells while using minimal sample/reagents. There was a drastic difference between transistor characteristics for all devices as a result of the binding event. Both buffer and human serum samples were detected with Microfluidic-based OECTs in a broad range of concentrations. Since accumulation-mode devices require less power and have higher current changes during binding, they outperform depletion-mode devices. Unlike electronic immunosensors that rely on reference electrodes or electroactive labels, our simple detection method does not require reference electrodes [42].

In order to synthesize organic TPE nanoparticles (NPs), a new supercritical antisolvent (SAS) process was developed with a microreactor. A model organic molecule, tetraphenyl ethylene (TPE), was solubilized in tetrahydrofuran (THF) [43], and supercritical carbon dioxide (sc-CO_2_) was used as an antisolvent. It is possible to obtain sizes below 15 nm using µSAS. Narrow dispersion size (±3 nm) was obtained. This technique is very useful for producing very small organic nanoparticles, paving the way for a wide range of practical applications [44].

**Scheme 2:**
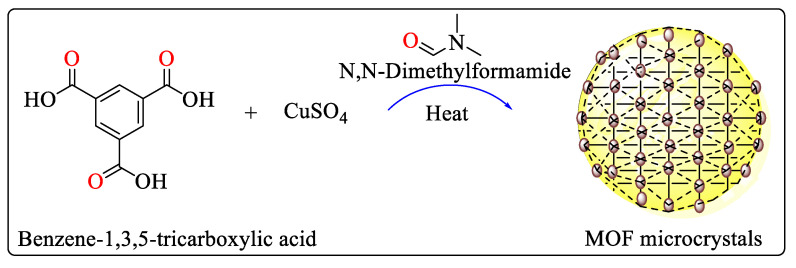
Microfluidic reaction to formulate MOF microcrystals.

Metal–organic framework microcrystals with HKUST-1 topology [45] of different sizes, shapes, and chemistry of metal clusters have been synthesized using the microfluidic system consisting of the microfluidic chip (MFC) and syringe pumps. The MFC contains two channels, the upper channel contained metallic salt (CuSO_4_, Cu(NO_3_)_2_ or Ni(NO_3_)_2_ preliminarily dissolved in *N*,*N*-Dimethylformamide), whereas the bottom channel was filled with the ligand (benzene-1,3,5-tricarboxylic acid or trimesic acid preliminarily dissolved in *N*,*N*-Dimethylformamide) shown in Scheme 2. The reaction rapidly started at the junction of two channels. For the temperature regulation, the MFC was placed on a refrigerant and a heating plate. Variations of the reaction temperature in the case of all three metallic salts used resulted in a change of the morphology of the obtained MOF microcrystals [46].

Previous studies have shown that a multifunctional nanoparticle drug delivery system (NDDS) is capable of delivering hydrophilic doxorubicin (DOX) and near-infrared photosensitizer dye IR780 simultaneously using a nanoscale zeolitic imidazolate framework-90 (ZIF-90) core and a SAD shell using a microfluidics-based approach. ZIF-DH obtained by IR780 has a 10-fold loading capacity over ZIF-90 obtained by conventional means. A spermine-modified acetalated dextran (SAD) shell could further enhance the pH-responsive release and prevent drug leakage in physiological solutions by improving the pH-responsive release performance of nanoscale ZIF-90. ZIF-90 is also capable of targeting tumors due to its conjugation of hyaluronic acid (HA) via amine groups on its SAD shell. A synergistic dual-mode chemo-photodynamic therapy using IR780/DOX@ZIF-DH was shown to be effective against cancer both in vitro and in vivo. As a result of this study, multiple problems encountered in nanoscale ZIF are simultaneously solved by combining intelligent polymer design and controllable microfluidics-based production. It is expected that this technology has great potential for cancer therapy clinical applications [47].

**Figure 1:**
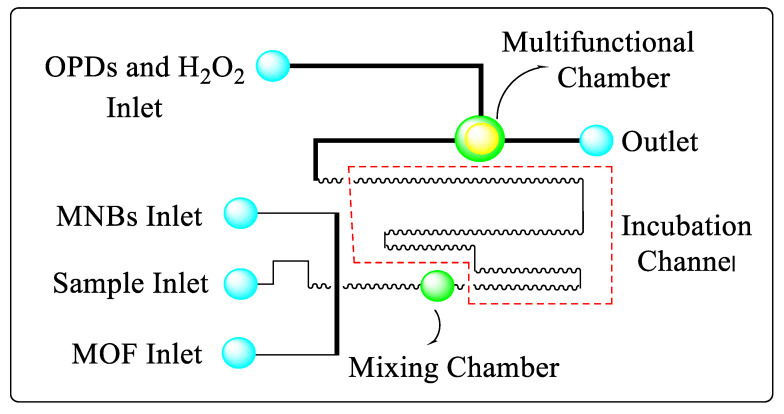
Diagram of the microfluidic chip to detect *Salmonella* typhimurium bacteria.

There are a number of foodborne pathogens, including *Salmonella*, that are found throughout the world. It can cause diarrhea, gastroenteritis, typhoid, and other symptoms when found in a wide range of foods. To prevent and control foodborne diseases, rapid and sensitive detection of *Salmonella* is essential [48]. In conventional methods, bacteria are mainly detected by culture, enzyme-linked immunosorbent assays (ELISAs) and polymerase chain reactions (PCRs) [49]. An automatic, rapid, sensitive and automated detection system for foodborne pathogens has been successfully developed using a microfluidic biosensor [50,51]. An MNB–*Salmonella*–MOF complex was formed using the immune magnetic nanobeads (MNBs) and the immune magnetic nanofoams (MOFs) to detect *Salmonella* typhimurium. As a result of the catalysis by the complexes, yellow 2,3-diaminophenazine (DAP) was produced by combining o-phenylenediamine with H_2_O_2_ as shown in Figure 1. The catalyst was then photographed using a narrow-band blue light. With greater sensitivity, the improved image processing was demonstrated to be effective for rapidly mixing the solutions in the vibrating mixer. Detection of *Salmonella* typhimurium at a concentration of 14 CFU/mL was achieved using the microfluidic biosensor, which integrated mixing, incubation, separation, catalysis, and detection. Despite its microfluidic design, this microfluidic biosensor does not cost more than USD 200. For ensuring food safety, it can be extended for in-field screening of other foodborne bacteria [52].

Approximately 30 thousand tons of cephalexin, which is a kind of *β*-lactam, are produced each year and the antibiotic sells for USD 15 billion annually [53]. An integrated microfluidic platform was developed to manufacture cephalexin continuously through an enzymatic reaction. Microspace is used on this platform as an excellent environment in which to perform continuous reaction–transport processes. This platform offers simultaneous synthesis and separation of reaction products in combination with an aqueous two-phase system (ATPS). It is screened for the composition of ATPS that allows the separation of the enzyme from the reaction product. In order to synthesize cephalexin, the bottom (salt) phase of ATPS is used as a reaction medium. During the kinetic regime, reaction conditions are set so that maximum cephalexin yields can be achieved. Afterward, slug-flow microfluidic platforms are used to test the enzyme recycle of cephalexin synthesis. A modular microfluidic system with a gravity settler and a microdialysis unit is utilized to continuously synthesize cephalexin [54]. 

**Scheme 3:**
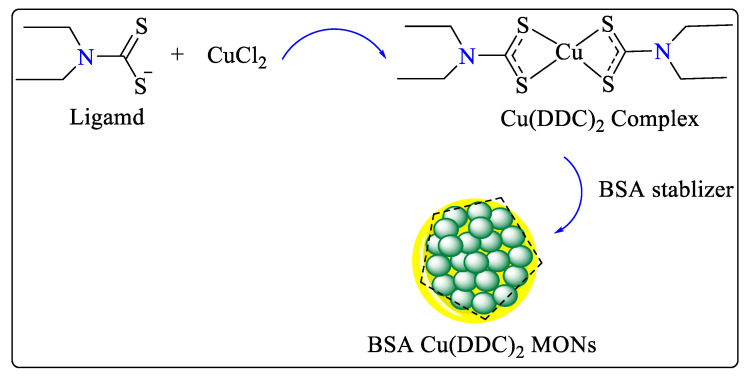
Synthetic reaction performed in microfluidic system to synthesize BSA Cu(DDC)_2_ MONs.

Metal–organic nanoparticles (MONs) were prepared from diethyldithiocarbamate copper Cu(DDC)_2_ using a Stabilized Metal Ion Ligand Nanocomplex (SMILE) technique [55]. In addition to being a superior yielding and high-concentration method, the SMILE method also requires a simplified process for formulation and preparation, and it has excellent formulation properties. The 3D-printed microfluidic device was used to further develop the SMILE technology for continuous production of bovine serum albumin Cu(DDC)_2_ MONs (Scheme 3). The microfluidic device was used to achieve precise mixing control, to easily scale up preparation for mass production, and to demonstrate great potential for treating breast cancer [56].

A microfluidic chip based on glass capillaries was used to produce solid–lipid nanoparticles (SLNs) for the first time. The current synthesis method showed several advantages over conventional bulk methods, which usually suffer from multiple preparation steps, low production rates and poor reproducibility, as well as being able to produce SLNs continuously with high yields, to be highly reproducible, and to be precisely controlled over their physical properties. For the purpose of testing the efficacy of SLN-based nano formulations in cancer therapy, sorafenib (SFN) and paclitaxel (PTX) were used as model drugs. As a result of the microfluidic production of SLNs, the drugs were encapsulated efficiently and loaded sufficiently to sustain a sustained release. In addition, fluorescence assisted imaging was used to confirm tumor penetration and cellular uptake as well as the anti-cancer efficacy of the drug-loaded SLN formulations [57]. 

**Figure 2:**
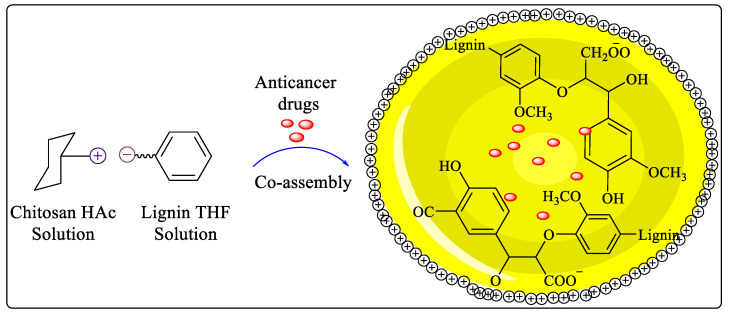
Microfluidic synthesis of Lig/Chi NPs.

A microreactor device was used for the successful preparation of lignin/chitosan pH-responsive polymer nanoparticles (Lig/Chi NPs) used for drug delivery. The electrostatic assembly of amino groups of chitosan and carboxyl groups of lignin formed Lig/Chi NPs during the mixing of positively charged chitosan and negatively charged lignin solutions in a microreactor. There is a positive charge on the surface of the nanoparticles, and they are very dispersible as shown in Figure 2. With the microreactor, lignin-based nanoparticles (LNP) suspensions with high stability and controlled size distributions can be produced with more uniform mixing and better control of the solvent/antisolvent ratio throughout the process. Lig/Chi NPs have been demonstrated as pH-responsive drug delivery carriers in these studies, and their production and application have reached a large-scale and commercial level [58].

**Figure 3:**
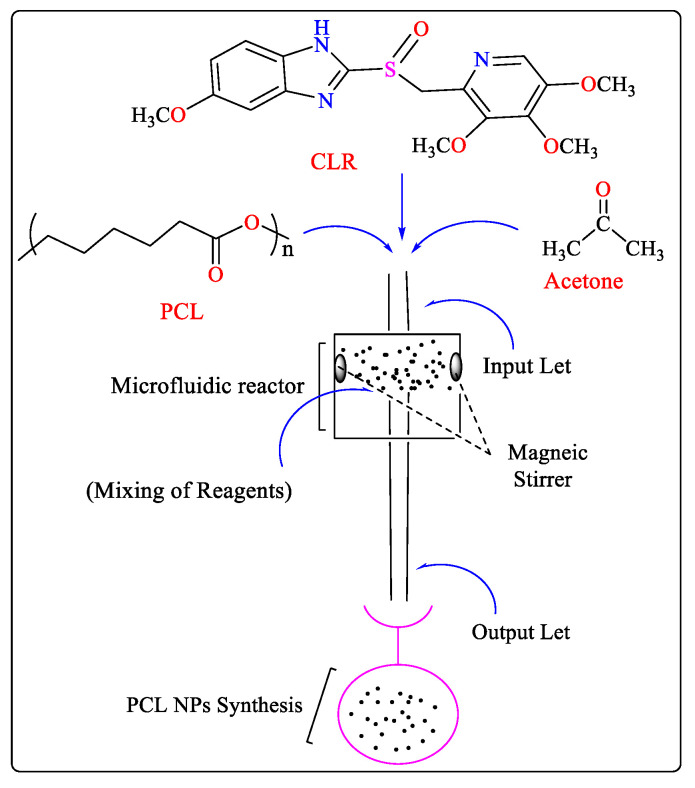
Square-shaped microfluidic reactor-based synthesis of PCL NPs.

Poly(ε-caprolactone) (PCL) is a polymer made from synthetic materials and has significant drug-loading capacity (LC), biodegradability, non-immunogenicity, and permeability, all of which improve the controlled release of drugs [59]. PCL nanoparticles (NPs) containing clarithromycin (CLR) have been synthesized using the microfluidic technique. To ensure the most homogeneous solution, 0.04 g of PCL granules were added to 20 mL of acetone, and the mixture was agitated for 1 h at 45 °C and 1 h at room temperature. Subsequently, CLR powder was dissolved in a cooled polymer solution in a tightly closed container. To produce NPs, distilled water was injected into the adjacent channels at a flow rate of 50 mL/h, whereas the PCL/CLR solution was injected into the middle channel at a rate of 2.5 mL/h. Subsequently, the two fluid flows were joined by a hydrodynamic flow-focusing event in the mixing region, where two liquid continuous phase streams surrounded the scattered phase stream and split it into droplets. To conduct additional analyses, the solution was lyophilized after it was removed from the outlet channel, after which the PCL NPs were formed (Figure 3). The resulting NPs were smaller, more uniform, and more stable than the NPs prepared using conventional methods [60].

### 2.2. Photocatalytic Applications

Synthetic organic photocatalytic chemistry is an effective method for easily producing both naturally occurring chemicals and compounds with highly complex structures under favorable circumstances. However, implementing a photochemical mechanism in an industrial process has proven challenging owing to scaling-up difficulties. With flow chemistry, it is possible to have more control over the reaction parameters, as well as higher reaction selectivity and reproducibility [61,62,63]. In contrast to conventional continuous-flow systems, droplet microfluidic technology uses multiple immiscible fluids to create a series of droplets that serve as analysis units. This avoids the issues of sample diffusion and cross-contamination, while further reducing the amount of reagent used. This method modifies fluids in microscale channels and offers a framework for several chemical synthesis approaches, leading to materials and molecules [64].

Metal catalysts and large quantities of hazardous organic solvents are required for the practical production of polyconjugate polymeric materials. Aqueous solutions containing micelle-forming agents can easily substitute for non-aqueous solvents, even when used for the manufacture of water-insoluble organics. Recently, the researchers described a selective metal-free photoinduced microfluidic system-based direct arylation procedure conducted in a laboratory setting with water, ambient temperature, and minimal competitive dehalogenation. The surfactant Kolliphor EL (K-EL) and a specially formulated photo redox mediator (S-PTh), which also functioned as a cosurfactant, partitioned numerous reactive species into different compartments of the association colloid formed in water, resulting in unexpected selectivity. The synthesized arylated compounds prepared by batch process had several drawbacks in terms of reagent amount, selectivity, reproducibility, and preparation time. To overcome these problems, the reaction was performed in a microfluidic reactor system and the results were surprising [65]. Table 1 shows the summary of role of microfluidic system in pharmaceutical industry.

**Figure 4:**
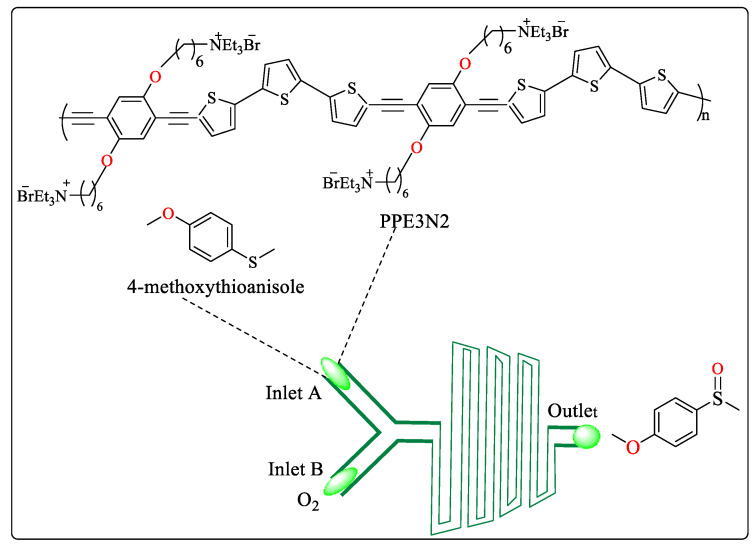
Illustration of PPET3-N2 as a photosensitizer to oxidize sulfides in microfluidic reactor.

Compared to conventional batch synthesis, microfluidic reactors have demonstrated considerable advantages in photocatalytic organic synthesis because they transfer heat and mass rapidly, have short molecular diffusion distances, are easy to control, and are light transplant [66,67,68]. Researchers reported a cationic poly(p-phenylene ethynylene terthiophene) (PPET3-N2) as a sensitizer for effective photocatalytic oxidation of a series of organic sulfides in microfluidic device (Figure 4). In the present study, photocatalytic oxidation of 4-methoxythioanisole was performed. The channel in the microfluidic reactor was filled with mixed solution of PPET3-N2 and 4-methoxythioanisole in methanol. In order to keep the microfluidic reactor in an oxygen rich environment, oxygen was delivered into another channel using a syringe pump [69]. Table 2 shows summarization of microfluidic system based photocatalytic synthesis.

### 2.3. Biochemical Applications

Several new methods have been demonstrated recently for the construction of multifunctional wound healing materials. As the substrate, a polyvinylpyrrolidone (PVP)-oriented membrane was spun by microfluidics to create an oriented microfiber membrane. A zeolitic metal–organic framework-8 at ascorbic acid (ZIF-8@AA) was used as a framework to load ascorbic acid in drug-delivery nanomaterials. Following microfluidic spinning, the PVP-oriented membrane showed robust antibacterial and drug release performance. Biocompatibility and hemocompatibility of the fabricated PVP membrane were excellent in vitro. The present work offers a method of constructing regular microfiber arrangements and allowing active materials to be loaded into substrates in the biomedical field for disease treatment [70]. 

An insulin delivery system based on a metal–organic framework (MOF) has been developed using microfluidics. Through a continuous-flow, microfluidic mixing system, ZIF-8 was synthesized with insulin and gold nanoparticles (AuNPs). To oxidize glucose molecules absorbed by porous ZIF-8, glucose oxidase mimicking the function of AuNPs was used. As glucose was oxidized inside the MOF, gluconic acid and hydrogen peroxide were produced. It is possible to use these synthetic bioactive MOFs to develop stimulus-responsive drug delivery systems and to exploit them for biosensing applications [71].

**Figure 5:**
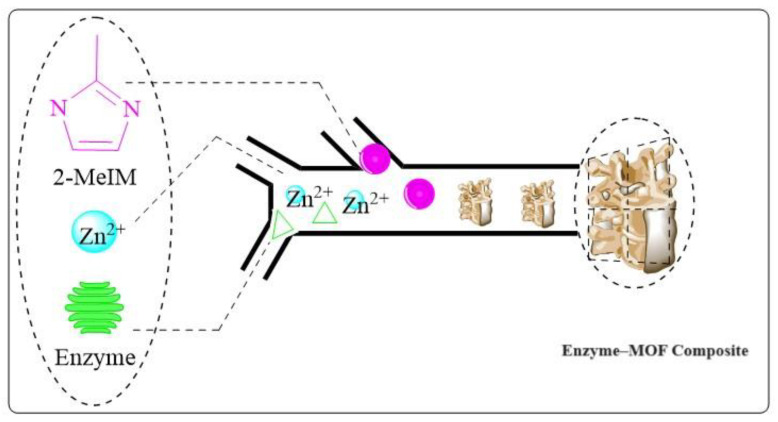
Microfluidic system-based synthesis of enzyme–MOF composites.

In Figure 5, enzyme–MOF composites were synthesized using a double-Y-shaped microfluidic channel. First, zinc ions (Zn^2+^) were mixed with 2-methylimidazole (2-MeIM), followed by protein. Through centrifugation and washing steps, the product was continuously collected from the outlet. As the ratio of reactants in the microchannel continuously changed, mesopores appeared in the resultant products. The multimodal distribution of pore sizes enabled enzymes to be immobilized while reducing resistance to mass transfer. As a result, enzyme–MOF composites prepared from conventional bulk solution synthesis are believed to have a nearly twofold higher enzymatic activity than enzyme–MOF composites. As a result of this defect-assisted synthesis, a new approach to enhancing enzyme–MOF composite activity has been proposed [72].

Through a microfluidic approach, researchers have developed BioZIF-8 MOFs that are aptamer-functionalized in a single step and using a single chip. Microfluidics enabled the encapsulation of nucleic acids, proteins, and small drug molecules while simultaneously functionalizing BioZIF-8 MOFs with an aptamer (RNA and DNA aptamers). It was found that neat BioZIF-8 MOFs had a much lower toxicity profile and were more effective at targeting lymph nodes and tumor masses than the aptamer-BioZIF-8 MOFs [73,74,75]. In this study, BioZIF-8 MOFs are prepared using a simple one-step and one-chip microfluidic method [76].

**Figure 6:**
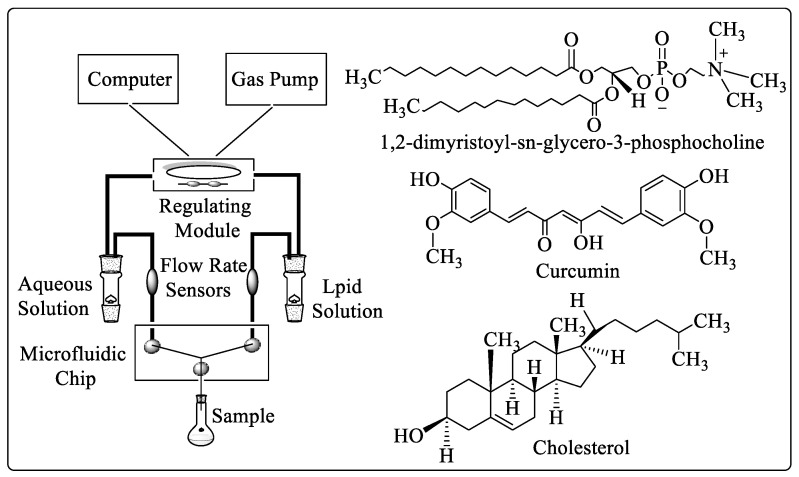
Microfluidic methodology for synthesis of curcumin-loaded liposomes.

It was confirmed that the microfluidic method was a simple and fast method for producing liposomes (Figure 6). A uniform size distribution was observed for curcumin-loaded liposomes and empty liposomes produced by microfluidics. Liposome production using LCD-printed microfluidic devices was studied for the first time in this study. It is necessary to consider scalability, process control, and multistep processes when fabricating microfluidic devices because traditional fabrication techniques suffer from many disadvantages, including multistep processing, costly facilities, low encapsulation efficiency, and low encapsulation efficiency, among others [77]. A novel method for preparing 1,2-dimyristoyl-sn-glycero-3-phosphocholine liposomes with cholesterol was developed by using commercial and 3D-printed microfluidic devices. The anticancer activity of curcumin produced by microfluidics has been reported to be enhanced when compared with other reported systems that encapsulate curcumin [70,78].

To scale-up the production of lipid nanoparticles (LNPs) and small interfering RNA (siRNA)-loaded LNPs, an integrated baffle device has been designed. By using a microfluidic stepwise post-treatment system, a seamless and simple manufacturing process could be achieved. It demonstrated high knockdown activity and low accumulation of siRNAs in mice hepatocytes when LNPs were delivered effectively. A microfluidic post-treatment method did not significantly affect siRNA delivery or LNP activity, according to scientists. The rapid ethanol dilution method, in comparison to conventional post-treatment methods, allowed greater precision in size control for LNPs. Integrated baffle devices can be used to produce more precise LNP-based nanomedicines under strict hygiene conditions, improving the efficacy of current nanomedicines and enabling high-throughput production to improve health outcomes [79,80].

Microfluidic chips can be used to automate and integrate DNA-based data storage by encapsulating DNA micro bibliographies within 10 min of encapsulation and extracting them within 5 min. By combining DNA microlibrary@MOFs with data-encoded DNA, harsh environments can be effectively handled. When stored at 25 °C, 50% relative humidity, and 10,000 lx of sunlight radiation, the encoded information can be read out perfectly after accelerated aging. As well as this, the library can be reproduced every time it is accessed so that target data can be retrieved in real-time via flow cytometry [81].

**Figure 7:**
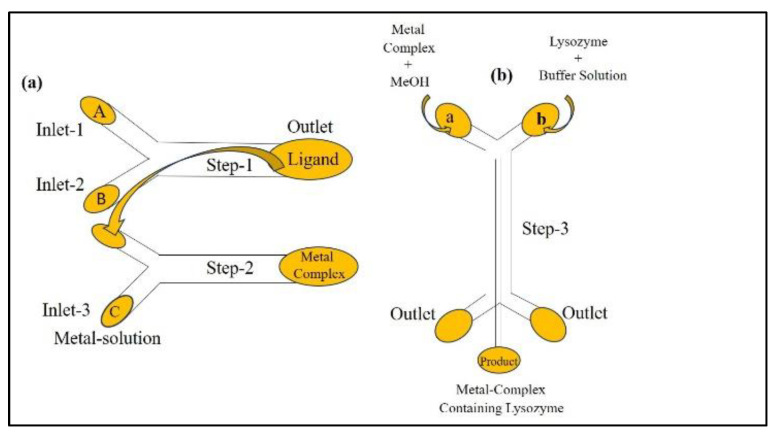
Microfluidic system-based synthesis: (**a**) Metal complex synthesized using Y-junction; (**b**) X-junction synthesis of a metal complex containing lysozyme. A = compound **1**, B = Compound **2** and C is metal solution.

In recent years, scientists have focused on building metal complexes with proteins that can be used as fuel cells or for drug development. Chemical synthesis has enabled the extensive use of microfluidic devices [82]. Several complexes comprising proteins have been developed using conventional-scale glassware [83]. The oxidative degradation of metal complexes by ambient oxygen is a major problem because the reaction time is prolonged at this scale. After synthesis, it is necessary to separate the complex from the unreacted compounds and organic solvents. Figure 7 depicts the microfluidic apparatus used to manufacture the chiral salen Mn(II) and Co(II) complexes with lysozyme because rapid reactions are possible using microfluidic chemical synthesis [6]. It was observed that the enclosed channel walls would reduce the influence of the atmosphere and that the reaction time would be lower at lower temperatures.

**Scheme 4:**
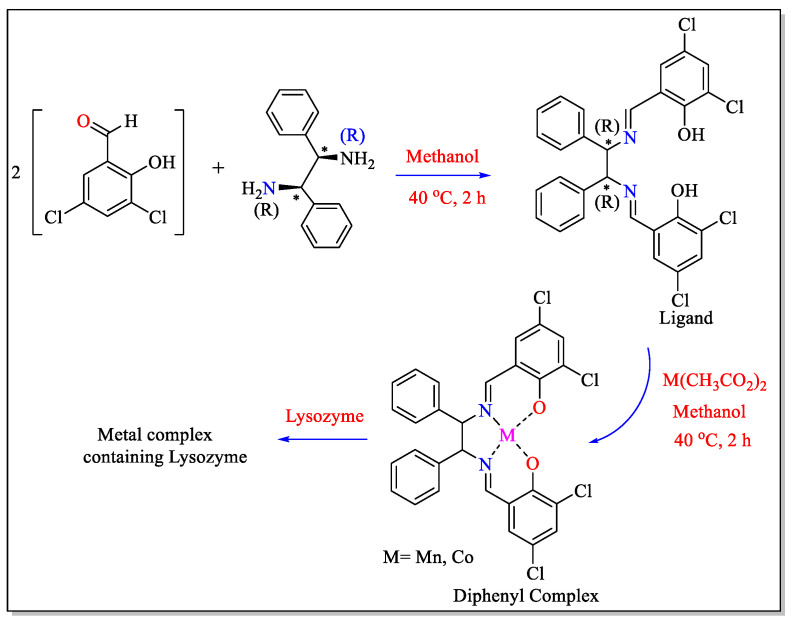
Synthesis of lysozyme-containing diphenyl complexes using conventional beaker method. * (Shows Chirality in a structure).

As shown in Scheme 4, in Step 1, methanol (50 mL) is dissolved dropwise to form a solution of (1*R*,2*R*)-(+)-1,2-diphenylethylenediamine (B). The mixture is then agitated at 40 °C for 2 h to produce a yellow solution of the ligand. Step 2 involves the formation of a metal complex from the ligand and Mn(II) or Co(II) acetate tetrahydrate (C), both of which are dissolved in methanol. Synthesis is performed without any treatment after Step 1, and the ligand inlet for Step 2 is connected to the outlet of Step 1, as shown in Figure 7a. The interaction between the Mn (II) complex and lysozyme in Step 3 produces Mn(II) and Co(II) complexes containing lysozyme. The X-junction apparatus employed in the experiment is shown in Figure 7b. To separate the unwanted products, this instrument incorporates a separation barrier. A sodium citrate buffer solution (pH 4.25) is mixed with the Mn(II) complex after slight dissolution in methanol. Lysozyme is mixed in a buffer solution, and the lysozyme solution is injected from inlets a and b in Figure 7b together with Mn(II) or Co(II) complexes, respectively [10]. Table 3 shows microfluidic system based Biochemical synthesis.

### 2.4. Fine Chemical Production

**Figure 8:**
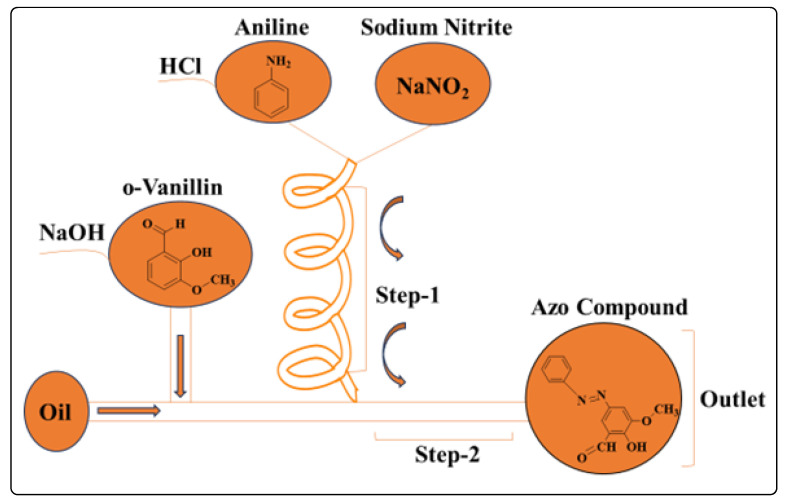
Microfluidic system for the synthesis of azo compounds.

Azo compounds are adaptable substances used in a wide range of products including batteries and anticancer medications and are typically produced via challenging solution-based processes [26,84]. To facilitate the chemical reaction, diazotization is typically performed in a highly concentrated aqueous HCl solution. The pH is brought back to neutral because excessive amounts of either alkaline or acidic reagents can cause product deterioration. However, these conventional solution-phase synthesis methods require challenging procedures involving metal catalysts, precise temperature, and environmental control. Consequently, a unique method for producing azo compounds known as microfluidics-based pH control, in which all of the difficult steps are completed in tiny droplets, has been developed. Using the microdroplet technique, the required azo molecule could be produced quickly, allowing the reaction to be conducted at ambient temperature (23 °C) as opposed to 0 °C and reducing the reaction time from 1 h to 3 s or less. Furthermore, pH control can be easily attained, and the reagent concentration can be lowered to one-tenth of what is typically required. In addition, the presented microfluidic device makes it simple to alter the reagent concentration by changing the rate at which it flows, and synthetic experiments may be conducted under various conditions (Figure 8).

**Scheme 5:**
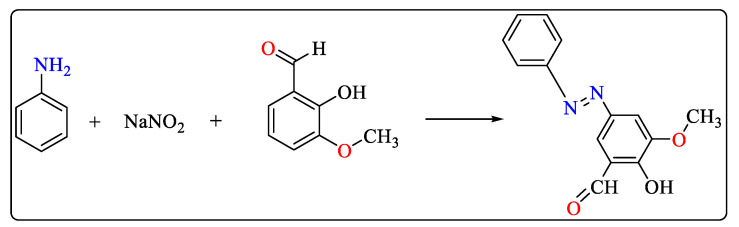
Reaction scheme for the synthesis of azo compounds.

Aniline, sodium nitrite, and o-vanillin were used to prepare azo compounds according to Scheme 5. Aqueous HCl and sodium hydroxide solutions were used to dissolve aniline and o-vanillin, respectively. To investigate their impact on the chemical reaction, the flow rate varied with pH and reagent concentration. After filtration, the azo compound was isolated [85].

**Figure 9:**
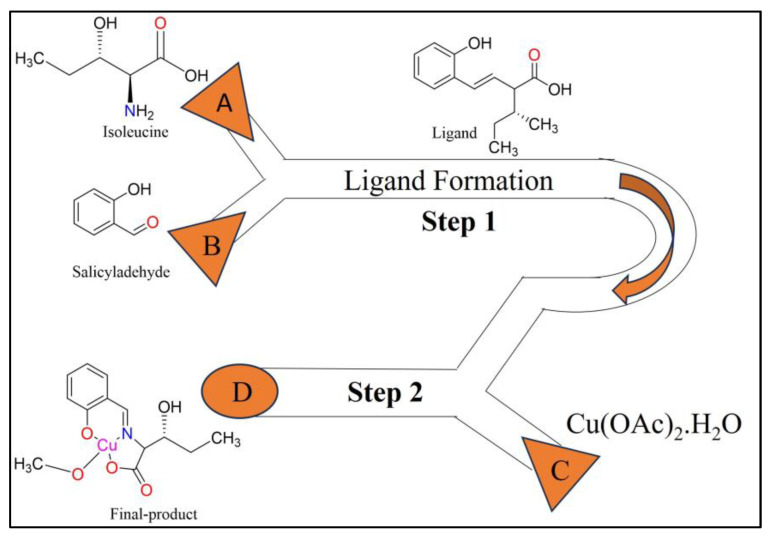
Microfluidic synthesis of Schiff base Cu(II) complex.

The two-step microfluidic system-based synthesis of a Cu(II) complex with a Schiff base is shown in Figure 9. Isoleucine and salicylaldehyde are injected from inlets A and B, respectively, at a rate of flow 5 L/min. The Schiff base ligand is produced in Step 1. Inlet C is used to induce the formation of a Cu(II) acetate dihydrate solution (20 mmol/L) at a flow rate of 10 L/min. The Cu(II) complex ligand is produced and removed as a solution from outlet D. Syringes and syringe pumps are used to inject the reactants dissolved in methanol into the inlets. This facilitates the precise control of the flow rate of the microfluidic device and the amount of reactants added [19].

**Scheme 6:**
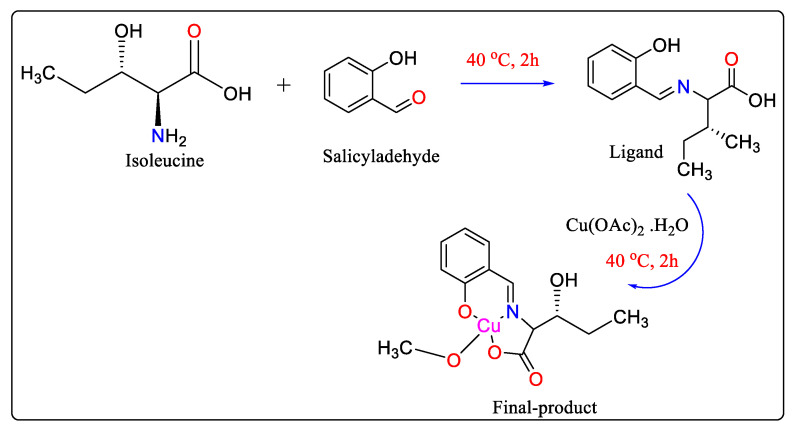
Conventional beaker-based synthesis of Schiff base Cu(II) complex.

Schiff bases are produced via the combination of amino and carbonyl groups containing multidentate ligands to synthesize highly significant complexes with metal ions. These compounds are used as polymer stabilizing substances [86], anti-corrosion [87], dyes, pigments, catalysts, antioxidants [88], carcinogens, and antimicrobial agents [89]. For the first time, microfluidic technology allowed for temperature-free synthesis of the Schiff base Cu(II) complex in 20 s, and the reaction performance was approximately 700 times greater than that of the synthesis utilizing the beaker [90] (Scheme 6).

**Scheme 7:**
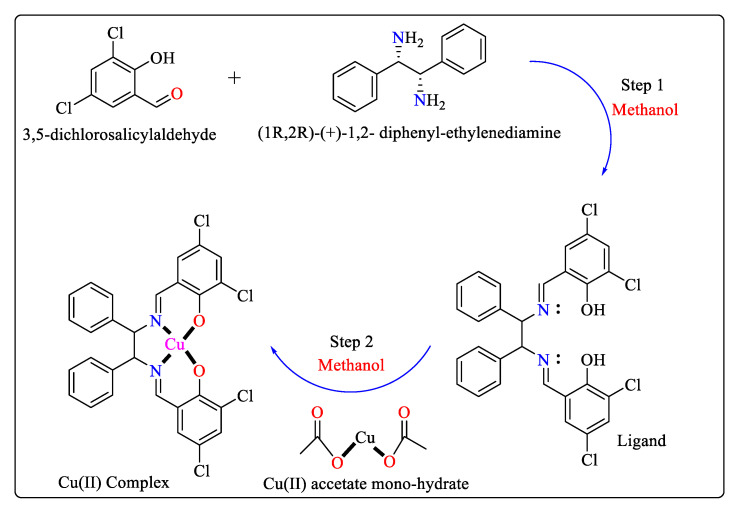
Schematic pathway for the synthesis of Cu(II) complex. Step 1 for the synthesis of ligands and Step 2 for the synthesis of metal complexes.

Another Cu(II) complex was produced like the one described above. As shown in Scheme 7, as a starting point, a ligand is prepared using 3,5-dichlorosalicylaldehyde and (1*R*,2*R*)-(+)-1,2-diphenyl-ethylenediamine in a beaker with methanol as the solvent. Next, a droplet-merging device is used to synthesize a copper complex using the ligand and Cu(II) acetate monohydrate. The ligand and Cu(II) acetate monohydrate are effectively combined during the merging stage in a methanol solution. Immediately upon their merging, the droplet interiors were transparent; however, a complex crystal started to emerge after 0.2 s. The synthesis is almost finished in one second at an ambient temperature below 25 °C, as opposed to two hours at 40 °C for the beaker-level work [5].

Covalent organic frameworks (COFs) are a new class of molecular materials that rely on the precise chemical fusion of organic subunits to form 2D or 3D permeable crystalline frameworks coupled by covalent bonds with deterministic control over porosity, composition, and topology [91,92,93,94]. The majority of recently developed COFs are based on self-condensation reactions with other boronic acids or boronic acids condensed with catechol. Solvothermal reaction conditions are required to produce porous crystalline materials. However, the limited chemical stability of these COFs, despite their thermal durability, severely restricts their use in various applications [95,96]. Researchers reported the rapid synthesis of a crystalline COF at room temperature as part of our efforts to overcome these limitations.

**Figure 10:**
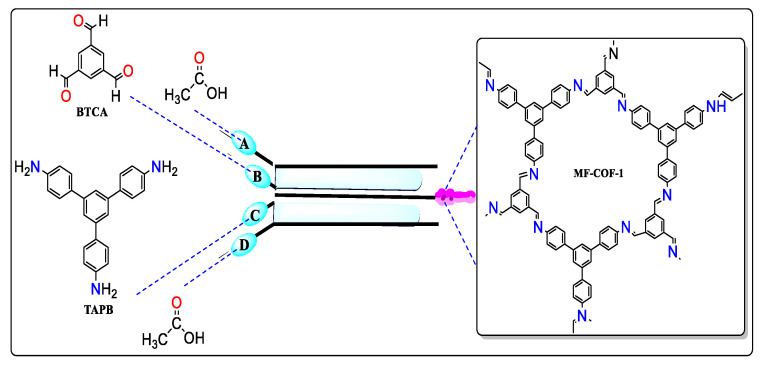
Microfluidic synthesis of MF-COF-1 at room temperature.

For the first time, an imine-based COF material (MF-COF-1) was developed under continuous microfluidic conditions. At atmospheric pressure and room temperature, MF-COF-1 could be manufactured in only a few seconds (~11 s). In a typical study, 1,3,5-benzenetricarbaldehyde (BTCA) and 1,3,5-tris(4-aminophenyl)benzene (TAPB) solutions with acetic acid were used to synthesize MF-COF-1. As shown in Figure 10, channels (A) and (D), are filled with purified acetic acid, whereas channels (B) and (C) are filled with BTCA and TAPB fluids, respectively. A syringe pump is used to inject the solution into the microfluidic device. Macroscopic fibers such as jelly are produced during the microfluidic production of MF-COF-1 and can be readily collected or placed on surfaces [97].

**Figure 11:**
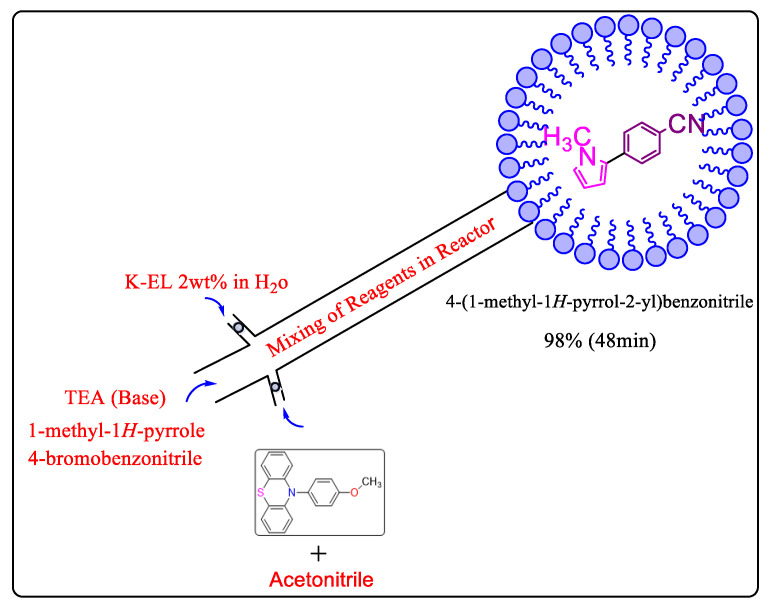
Microfluidic-based synthesis of pyrrole.

**Scheme 8:**
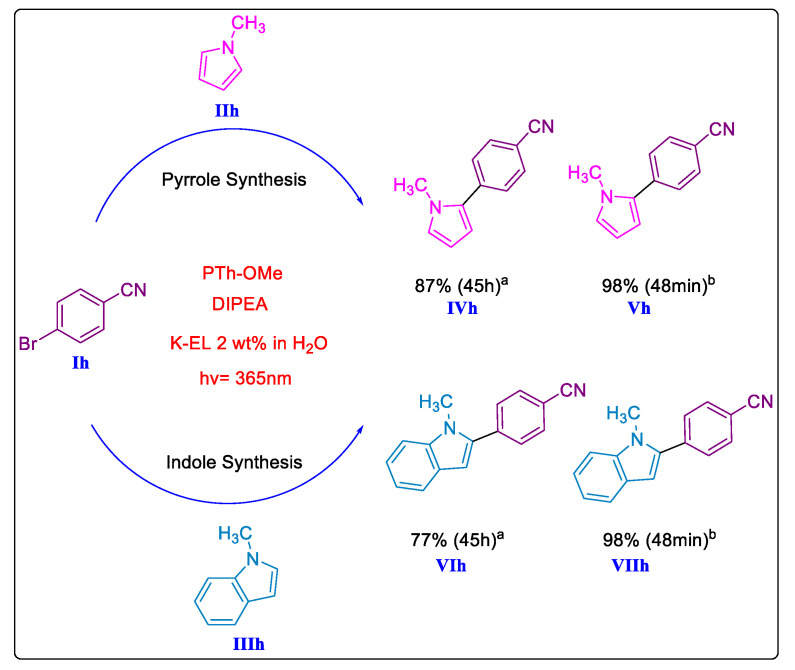
(a) Synthesis through batch processing; (b) synthesis through microfluidic reactors using TEA base.

To exploit the known catalyst 10-(4-methoxy)phenyl-10H-phenothiazine (PTh-OMe), researchers arylated N-methyl pyrrole with ethyl-4-bromobenzoate. The pyrrole and indole couplings reported here were performed by preparing a stock solution of ethyl 4-bromobenzonitrile (Ih), N-methylpyrrole (IIh), and N, N-diisopropylethylamine (DIPEA), and the PTh-OMe catalyst was dissolved in 15 mL of acetonitrile. After a suitable period, 12 mL of this solution was recirculated inside the reactor while being exposed to radiation. The resulting solution was emulsified by sonication in an ultrasonic bath for 15 min with 15 mL of a 2 wt% K-EL surfactant in an aqueous solution. This emulsion was then injected into the microreactor channels of the microfluidic system under radiation at a flow rate of 0.1 L/min while put under light for 100 min. To maximize the mixing, thermal exchange, and uniformity, the reaction mixture was pushed through microchannels with a high surface-to-volume ratio. By parallelizing the reaction flasks, microfluidic reactors can drastically increase efficiency without reducing the usability of synthetic materials. To demonstrate the selectivity of the process, decreasing the excess of the arylation partner from the usual 20–50 fold to 5.5 fold as compared to the batch process and the reaction time for reagent coupling from hours to minutes by replacing the base DIPEA with triethanolamine (TEA) [65]. A similar procedure was used for the synthesis of indole using the microfluidic system in excellent yield (Scheme 8). The microfluidic system-based synthesis of pyrrole is shown in Figure 11.

Using femtosecond laser micromachining, microfluidic chips (MFCs) consisting of centimeter-level 3D channels with large volume and high density were developed for timesaving, economically viable, and hazard-free flow synthesis. Its advantages have been demonstrated by forming aryldiazonium salts in situ and borylating them with bis(pinacolato)diboron. The 3D MFC-based flow synthesis technology we developed offers several important advantages, such as (1) altering the reaction temperature from an ice bath to room temperature; (2) reducing the residence time by 10 times; and (3) significantly increasing yields, as several aryl-boronates were produced with higher yields than traditional batch processes. It is therefore anticipated that a novel, simplified flow synthetic protocol will be developed for green organic synthesis using MFCs [98].

**Scheme 9:**
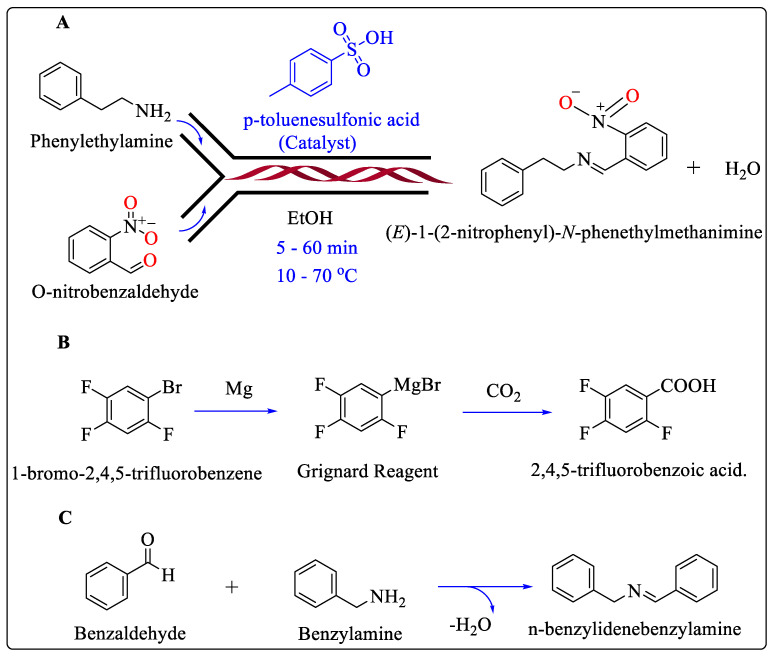
(**A**) The model reaction performed in a microfluidic system, (**B**) microfluidic reaction to convert CO_2_ into 2,4,5-trifluorobenzoic acid, and (**C**) synthesis of *n*-benzylidene benzylamine.

A microfluidic system was discovered here that automates chemical reaction screening and optimization inside microliter liquid droplets. Droplet “micro-reactors” are generated, merged, and flowed, and reaction conditions, including reagent volumes, temperature, and time, are precisely controlled by the system. Input parameters can be thoroughly monitored by allowing multiple reaction conditions to be screened simultaneously due to the high level of control coupled. Furthermore, there is a remarkable reduction in reagent consumption. The researcher demonstrated the use of a microfluidic platform to screen a model imine formation-(*E*)-1-(2-nitrophenyl)-*N*-phenethylmethanimine using ethanol as a solvent through a condensation of o-nitrobenzaldehyde and phenylethylamine. Tests were conducted using microfluidics to determine (i) the ratio of reagents (aldehyde and amine), (ii) the temperature, (iii) the reaction time, as well as (iv) the effect of *p*-toluenesulfonic acid as a catalyst (Scheme 9A) [99].

Microfluidics technique utilizes femtosecond laser-fabricated 3D microfluidic chips (MFCs). Using a packed-bed reactor and CO_2_ gas–liquid carboxylation, this innovative approach enhances the efficiency of CO_2_ gas–liquid carboxylation. Using CO_2_ and Grignard reagents under mild conditions (Scheme 9B), this platform demonstrated ingenuity, simplicity, efficiency, and cost-effectiveness in enabling fluorinated aryl carboxylic acids to be synthesized. It is important to note that the 3D MFCs integration and automatic operation not only enhances productivity and flexibility, but also ensures utmost safety when creating continuous mass production [100].

According to Scheme 9C, benzaldehyde and benzylamine were used in the microfluidic synthesis of *n*-benzylidene benzylamine. It is a highly industrially relevant approach, since fluctuations in concentration, an incorrect dosage, or a loss of temperature control may not always be prevented in real industrial settings [101].

In 1907, phenol and formaldehyde were combined to create the first thermosetting resin, known as phenolic resin [102]. After more than a century of study and commercialization, the average annual worldwide production of phenolic resins is six million tons [103]. The long-term thermal, chemical, and mechanical durability, exceptional strength, excellent insulating abilities, and superior char yield properties of phenolic resins are widely recognized because of their significant cross-linking with aromatic dense structure [104]. In conventional synthesis techniques, formaldehyde and phenol are primarily used to produce phenolic resins at certain temperatures and pH levels, in which specific catalysts are frequently needed. Additionally, conventional synthesis is typically performed in a bulk system, which results in a significant number of side-reaction products owing to the loss of precise control over the reaction conditions. In recent years, microfluidics has become a unique technique for producing a range of phenolic resins with relatively small quantities of reagents, according to a previously reported structure [105,106,107,108].

**Figure 12:**
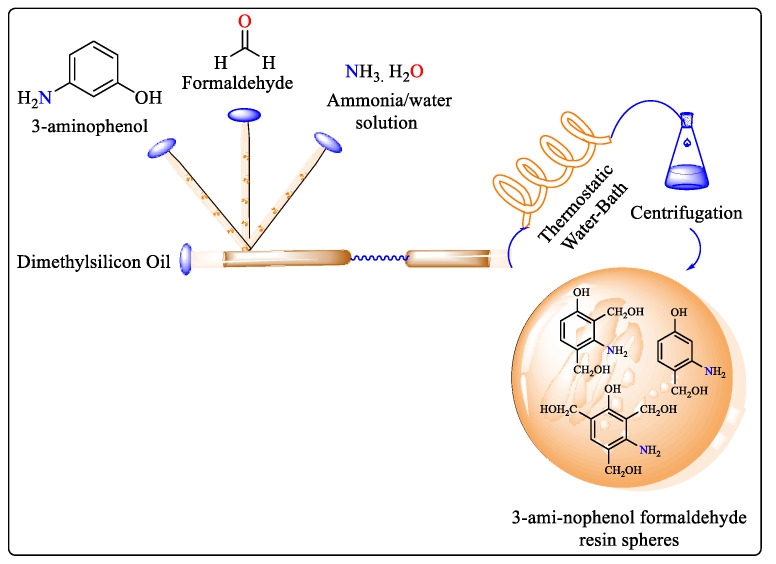
Microfluidic preparation of phenolic resins.

To prepare these solutions, 3-aminophenol, formaldehyde, and ammonia were dissolved in water. As a continuous phase, dimethyl silicone oil was injected at various Span 80 mass ratios. The prepared solutions were placed separately in syringes and delivered using syringe pumps that controlled the flow rate. The droplets were formed at the intake channel intersection. As they travel along the winding channel, the chemicals inside the channel mix rapidly. A tube with a helical structure that connects to the channel outlet is submerged in a water bath with a thermostat, as shown in Figure 12. The residence time is controlled by adjusting the overall length of the helical tube according to the total flow rate. Therefore, the chemical reaction is stopped promptly, and the products are removed in a conical flask containing an ice-water bath. Subsequently, alcohol/water rinsing, and rapid centrifugation are performed to separate and purify the product. The ideal residence period and temperature for the synthesis are approximately 270 s and 80 ℃, respectively [109]. Table 4 shows the summarization of microfluidic system-based fine chemical synthesis.

## 3. Conclusions

Various organic compounds, including tiny molecules and polymers with a narrow range of sizes, can be produced through microfluidic synthesis by properly mixing or focusing the reagents, component flows, and consistent morphology with superior thermal, optical, electrical, and magnetic properties. The application of microfluidic systems in the manufacture of organic compounds has been thoroughly studied over the past few years. The findings of this review demonstrate the immense potential of organic substances produced on such platforms. Despite significant recent progress, more research on the interface between microfluidic innovation and organic compound layout is still required to fully exploit the potential of microfluidic synthesis for the development of novel materials that are useful for biomedical, biosensing, and ecological engineering applications.

## Figures and Tables

**Table 1:** Summarization of microfluidic system based pharmaceutical synthesis.

Experiments	Type of Reaction	Advantages	Pharmaceutical Applications
Electrosynthesis of nematic liquid crystal compounds	Microfluidic redox-neutral molecular diffusion	Enhanced reactivity, Handle several samples simultaneously over a long period, Rapid molecular diffusion	Facilitates selective transformation
Detect amyloid-β protein in human serum	Microfluidic organic electrochemical transistors reaction	Reduced analytical time, Minimal sample, Not required reference electrodes	Cure Alzheimer’s disease
Organic TPE nanoparticles (NPs)	Microreactor based antisolvent process	Possibility to obtain sizes below 15 nm	Imaging, biosensing and medicine
MOF microcrystals with HKUST-1	Continuous flow synthesis in a microfluidic chip	2 times higher loading capacities than a solvothermally synthesized compound	Utilize them as drug delivery systems
Zeolitic imidazolate framework-90 (ZIF-90) core and a SAD shell	Microfluidics-Assisted Surface Trifunctionalization	10-fold loading capacity over ZIF-90 obtained by conventional means, prevent drug leakage	Cancer therapy, clinical applications
2,3-diaminophenazine (DAP) production	Used microfluidic biosensors	Greater sensitivity, improved image processing. rapid mixing	In-field screening of foodborne pathogens
Cephalexin is synthesized by penicillin acylase	Microfluidic aqueous two-phase system	High affinity, allows simple enzyme recycle and addition of fresh reactants.	Used as antibiotic
Solid-Lipid Nanoparticles (SLNs)	Oil-in-water homogenization microfluidic process	High yields and highly reproducible	Good encapsulation efficiency and loading degree of the drugs
PCL nanoparticles containing clarithromycin	Microfluidic hydrodynamic flow focusing reaction	NPs were smaller, more uniform, and more stable than the NPs prepared using conventional methods	Significant drug-loading capacity, biodegradability, non-immunogenicity
Bovine serum albumin production	Scalable continuous microfluidic assisted SMILE technology	Precise mixing control, easily scale up preparation for mass production	Potential for treating breast cancer

**Table 2:** Summarization of microfluidic system based photocatalytic synthesis.

Experiments	Type of Reaction	Advantages	Photocatalytic Applications
Arylated compounds	Photoredox reaction	Improves the reaction time, without eroding the yields and selectivity	Carrying out photoredox direct arylation couplings
Cationic poly(p-phenylene ethynylene terthiophene)	Photocatalytic oxidation	Transfer heat and mass rapidly, have short molecular diffusion distances and easy to control	Used as a sensitizer for series of organic sulfides

**Table 3:** Summarization of microfluidic system based Biochemical synthesis.

Experiments	Type of Reaction	Advantages	Biochemical applications
A zeolitic metal-organic framework-8 at ascorbic acid	Electrostatic hydrogen bonding, microfluidic spinning	Excellent biocompatibility and hemocompatibility	Construction of multifunctional wound healing materials
Insulin- and AuNP-encapsulated zeolitic imidazolate framework-8	Continuous-flow, microfluidic oxidation system	Due to presence of glucose stimulus for insulin release	Drug delivery, biosensing applications
Enzyme-MOF composites	Microfluidic laminar flow	Reducing resistance to mass transfer	Maintaining the protection to the enzymes
BioZIF-8 MOFs with an aptamer	Electrostatic one chip microfluidic reaction	Lower toxicity and more effective at targeting lymph nodes and tumor mass	Encapsulation of nucleic acids, proteins, and small drug molecules
Curcumin-loaded Liposomes	Microfluidic polymerization	Controlling the channel length, flow rates, composition, pH and temperature	Anticancer activity
Small interfering RNA-loaded LNPs	Continuous-flow, microfluidic system	High knockdown activity and low accumulation	Used for the treatment of various cancers
Chiral salen Mn(II) and Co(II) complexes with lysozyme	Microfluidic oxidation reaction	Reaction time would be lower at lower temperatures	Used in drugdiscovery or fuel cells

**Table 4:** Summarization of microfluidic system-based fine chemical synthesis.

Experiments	Type of Reaction	Advantages	Fine chemical Applications
Azo compounds synthesis	Diazotization reaction	pH control, short reaction time, at room temperature	Batteries and anticancer medications
Cu(II) complexes with a Schiff base	Laminar flow microfluidic reaction	Lower reagent quantity, precise control of the flow rate, reaction performance 700 times greater than conventional method	High biological activity and catalytic function, anti-corrosion, dyes, pigments, antioxidants, carcinogens, and antimicrobial agents
Imine-based COF material (MF-COF-1)	Self-condensation reactions,	At atm pressure and room temperature, compound manufactured in only a few seconds	Direct drawing of objects on surface
Arylated N-methyl pyrrole with ethyl-4-bromobenzoate	Microfluidic arylation reaction	Provide high surface-to-volume ratio	Polymer chemistry, biomedical science, pharmacy, isolation, and photochromism
Phenolic resins	Oil in water microfluidic reaction	Controlled residence time and flow rate	Thermosetting resin
Continuous CO_2_ transformation in multiphase flow	Gas-liquid carboxylation	High yield and short reaction time	Replacing the use of fossil fuel resources
Imine formation-(*E*)-1-(2-nitrophenyl)-*N*-phenethylmethanimine	Microfluidic condensation reaction	Low ratio of reagents, temperature, and reaction time	Dying, catalysis, paper industry

## Data Availability

No new data were created or analyzed in this study. Data sharing is not applicable to this article.
